# Reconsideration of the DTI-ALPS index as a glymphatic surrogate: disease-specific findings in small vessel disease due to cerebral amyloid angiopathy or arteriolosclerosis

**DOI:** 10.3389/fnagi.2026.1933808

**Published:** 2026-09-22

**Authors:** Yaping Zhou, Xingqi Pan, Ziyi Xia, Yuhui Sha, Gan Sun, Fei Han, Ming Yao, Lixin Zhou, Liyun Tu, Feng Feng, Joanna M. Wardlaw, Yi-Cheng Zhu, Jun Ni

**Affiliations:** 1 State Key Laboratory of Complex Severe and Rare Diseases, Department of Neurology, Peking Union Medical College Hospital, Chinese Academy of Medical Sciences and Peking Union Medical College, Beijing, China; 2 School of Artificial Intelligence, Beijing University of Posts and Telecommunications, Beijing, China; 3 National Infrastructures for Translational Medicine, Peking Union Medical College Hospital, Institute of Clinical Medicine, Theranostics and Translational Research Center, Chinese Academy of Medical Sciences and Peking Union Medical College, Beijing, China; 4 State Key Laboratory of Complex Severe and Rare Diseases, Department of Radiology, Peking Union Medical College Hospital, Peking Union Medical College and Chinese Academy of Medical Sciences, Beijing, China; 5 Institute for Neuroscience and Cardiovascular Research, UK Dementia Research Institute Centre, BHF-UK DRI Centre for Vascular Dementia Research, Row Fogo Centre for Research into Aging and the Brain, University of Edinburgh, Edinburgh, United Kingdom

**Keywords:** cerebral amyloid angiopathy, cerebral small vessel disease, diffusion tensor imaging, glymphatic system, NeuroImage

## Abstract

**Objectives:**

To explore the disease-specific performance of diffusion tensor imaging analysis along the perivascular space (DTI-ALPS) index in cerebral amyloid angiopathy (CAA) and arteriolosclerosis CSVD (aCSVD).

**Methods:**

We retrospectively enrolled probable CAA or aCSVD patients who underwent 5.0T MRI. Disease-specific DTI-ALPS characteristics and the correlations with clinical, imaging markers, and cognition were evaluated. Regression models were applied according to outcome distributions. Multiple comparisons were controlled using the Benjamini–Hochberg false discovery rate (FDR) procedure.

**Results:**

A total of 134 participants (56 probable CAA and 78 arteriolosclerosis, mean age 64.9 ± 10.0 years, 64.9% males) were enrolled. DTI-ALPS index was significantly lower in aCSVD than in CAA (*p* < 0.001). After adjustment for age and sex and FDR correction, no clinical factors remained significantly associated with DTI-ALPS index. In aCSVD, DTI-ALPS was significantly associated with cognitive performance, whereas these associations did not reach statistical significance in CAA. Across both groups, DTI-ALPS was significantly associated with white matter hyperintensity volume, skeletonized fractional anisotropy, skeletonized mean diffusivity, and peak width of skeletonized mean diffusivity (all FDR-adjusted *p* < 0.05). Associations with PVS burden and established CAA imaging markers did not reach statistical significance.

**Conclusion:**

DTI-ALPS index is heterogenous across distinct angiopathies but consistently associated with white matter microstructural damage. These subtype-specific findings question the relevance of DTI-ALPS index and caution against using DTI-ALPS index as a universal glymphatic surrogate.

## 1 Introduction

The glymphatic system (GS) has been proposed as a brain-wide perivascular transport pathway that facilitates cerebrospinal fluid (CSF) influx, interstitial fluid (ISF) exchange, and metabolic waste clearance within the central nervous system (Iliff et al., 2012). Through periarterial influx and perivenous efflux pathways, the GS is thought to contribute to the clearance of neurotoxic metabolites, including amyloid-β (Aβ), thereby maintaining cerebral metabolic homeostasis (Iliff et al., 2012). Increasing evidence suggests that glymphatic dysfunction may be associated with the pathophysiology of multiple neurological disorders, including Alzheimer’s disease (Tang et al., 2026; Taoka et al., 2017; Xu et al., 2023), Parkinson’s disease (Lv et al., 2025), demyelinating disease (Zhang et al., 2025), and cerebral small vessel disease (CSVD) (Xu et al., 2023). However, reliable *in vivo* assessment of glymphatic function in humans remains challenging. Direct evaluation of glymphatic clearance using contrast-enhanced magnetic resonance imaging (MRI), including intrathecal or intravenous gadolinium-based approaches, provides valuable insights into CSF-interstitial exchange and meningeal lymphatic drainage (Ringstad et al., 2017; Sennfält et al., 2023). However, the invasiveness and complexity of the techniques limited the implementation in routine clinical settings. Enlargement of perivascular spaces (PVS), as the structural basis for GS, is commonly regarded as an indirect marker of impaired glymphatic clearance and is associated with CSVD severity (Solé-Guardia et al., 2025). However, quantitative evaluation of PVS remains challenging because of their small size and limited visibility on conventional MRI (Solé-Guardia et al., 2025). Currently, validated surrogate neuroimaging markers of glymphatic function remain limited.

Recently, the diffusion tensor image analysis along the perivascular space (DTI-ALPS) index was proposed as a non-invasive imaging surrogate for glymphatic function (Taoka et al., 2017). The DTI-ALPS index was designed to quantify the diffusivity around penetrating vessels within the regions of interest (ROIs). The ROIs are strategically placed in the periventricular white matter, where the projection and association fibers were supposed to run perpendicular to the paraventricular veins and perivenous spaces (Taoka et al., 2017). Based on this geometric assumption, DTI-ALPS index was designed to minimize the influence of white matter fiber tracts and to isolate diffusivity potentially related to perivascular fluid movement (Taoka et al., 2017). A reduced DTI-ALPS index was considered indicative of impaired glymphatic function. Owing to its accessibility and non-invasive nature, DTI-ALPS index has been widely used in studies investigating glymphatic dysfunction across neurological diseases (Lv et al., 2025; Xu et al., 2023; Zhang W. et al., 2021; Zhang et al., 2025).

However, despite its widespread use, the biological validity of DTI-ALPS index as a glymphatic marker remains incompletely understood (Ringstad, 2024; Taoka et al., 2024). Since the underlying mechanisms of GS impairment vary across diseases, the resulting patterns of glymphatic impairment may also differ. However, the performance of DTI-ALPS index across different pathological etiologies remains unclear. Moreover, previous studies reported inconsistent associations between DTI-ALPS index and PVS burden, which has traditionally been considered a structural marker of glymphatic dysfunction (Solé-Guardia et al., 2025; Xu et al., 2022; Zhang W. et al., 2021). In contrast, DTI-ALPS index has shown relatively consistent associations with white matter hyperintensity (WMH) (Xu et al., 2022; Zhang W. et al., 2021). Moreover, recent methodological studies suggest that DTI-ALPS index measurements may be substantially influenced by white matter geometry and axonal organization rather than reflecting glymphatic transport directly (Mossige et al., 2026; Schilling et al., 2025; Taoka et al., 2024; Wright et al., 2024). Because the ROIs used in DTI-ALPS index calculations are small and confined to deep white matter, DTI-ALPS index is more likely to reflect localized vascular pathology rather than global glymphatic dysfunction. Given the spatial heterogeneity of vascular involvement across diseases, DTI-ALPS index performance may vary accordingly. Therefore, whether DTI-ALPS index effectively reflects glymphatic dysfunction or instead predominantly captures disease-related white matter microstructural injury remains to be elucidated across different angiopathies.

Cerebral amyloid angiopathy (CAA) and arteriolosclerosis CSVD (aCSVD, also called age-related or vascular risk factors related CSVD) represent two predominant subtypes of CSVD with distinct pathological features and spatial patterns of vascular involvement (Pantoni, 2010). CAA is characterized by pathological Aβ deposition that predominantly involves cortical and leptomeningeal vessels and is closely linked to impaired perivascular amyloid clearance pathways (Charidimou et al., 2017). In contrast, aCSVD primarily affects deep perforating arterioles and is associated with chronic hypoperfusion, diffuse white matter injury, and vascular risk factors (Pantoni, 2010). Therefore, if DTI-ALPS index predominantly reflects glymphatic dysfunction, a more pronounced reduction would be expected in CAA, a prototypical disorder of impaired GS clearance. Conversely, if DTI-ALPS index is driven predominantly by localized white matter microstructural injury, stronger alterations would be anticipated in aCSVD, which is characterized by severe white matter damage. However, the performance difference of DTI-ALPS index between CAA and aCSVD remained unclear.

Consequently, the present research aimed to explore the biological mechanisms of DTI-ALPS index by characterizing DTI-ALPS index patterns in the two distinct angiopathies. We further investigated associations of DTI-ALPS index with quantified PVS burden, white matter microstructural injury, and cognitive impairment across CSVD subtypes. This study may therefore help clarify the biological interpretation of DTI-ALPS index and its validity as a surrogate imaging marker of glymphatic dysfunction in CSVD.

## 2 Materials and methods

### 2.1 Study population

Patients were retrospectively identified from the CSVD cohort at Peking Union Medical College Hospital between September 2022 and November 2025 (Supplementary Figure 1). The CSVD cohort is an ongoing prospective research cohort that recruits patients presenting with neurological complaints and attending the Department of Neurology. The detailed inclusion and exclusion criteria for the CSVD cohort were described in our previous study (Zhou et al., 2026). For all eligible participants in the CSVD cohort, 5.0T MRI examinations were performed regardless of prior clinical or imaging data. The present study enrolled patients who underwent 5.0T MRI examinations and were diagnosed with probable CAA or aCSVD. The probable CAA was diagnosed according to the Boston v1.5 criteria (Linn et al., 2010) because the CSVD cohort was designed and initiated prior to the release of the Boston v2.0 criteria. The Boston v1.5 criteria were consistently applied to maintain methodological consistency across the cohort. The aCSVD was determined if patients showed ICH in the deep area or CMBs that were strictly deep or mixed-distribution, without lobar ICH or strictly lobar CMBs (Zhou et al., 2026). Patients were excluded if they presented (1) with other CSVD etiologies or an inconclusive etiology, (2) without 5.0T MRI results or with unqualified imaging, or (3) with missing crucial clinical or demographic data.

Informed consent was obtained from all the patients before their participation. The Ethics Committee of the Peking Union Medical College Hospital approved the study (JS-1280). The research was conducted in accordance with the Declaration of Helsinki.

### 2.2 Clinical evaluation

A standardized, structured case report form was used to collect clinical and demographic data. Vascular risk factors were recorded, including hypertension, diabetes, hyperlipidemia, smoking, and alcohol use. Cognitive function was evaluated by trained neurologists blinded to clinical and imaging data using the Montreal Cognitive Assessment (MoCA) and the Mini-Mental State Examination (MMSE). The MMSE and MoCA scores range from 0 to 30, with lower scores indicating severe cognitive impairment. The MoCA scores were education-adjusted with one additional point assigned to participants with ≤12 years of education according to the standard MoCA scoring procedure.

### 2.3 Neuroimaging acquisition

All participants underwent 5.0T MRI examinations (uMR Jupiter, Shanghai United Imaging Healthcare Co., Ltd.). The scanning sequences included 3D T1-weighted imaging (T1WI), 3D T2-weighted imaging (T2WI), 3D Fluid-Attenuated Inversion Recovery (FLAIR), susceptibility-weighted imaging (SWI), and diffusion tensor imaging (DTI). The DTI data were acquired using a spin-echo echo-planar imaging (SE-EPI) sequence with anterior-posterior (AP) phase encoding. The AP diffusion acquisition used a two-shell scheme comprising 3 b = 0 volumes and 32 hemispherically distributed diffusion-encoding directions at each non-zero b-value (b = 1,000 and 2,000 s/mm^2^). An additional 2 b = 0 volumes with reversed phase encoding (PA) were acquired for susceptibility-induced distortion correction. Total acquisition time was approximately 8 min 59 s for the AP sequence and 1 min 24 s for the PA sequence. The detailed parameters for the other sequences were summarized in Supplementary Table 1.

### 2.4 Automatic DTI-ALPS index calculation

Our present study adopted the previously validated automated DTI-ALPS index calculation method (Li W. X. et al., 2024), thereby minimizing manual intervention and improving reproducibility (Figure 1A). In our previous study (Li W. X. et al., 2024), this automated approach showed good agreement with manual calculation, with an intraclass correlation coefficient (ICC) of 0.81, supporting its reliability and reproducibility. The pipeline was implemented in Bash using the FMRIB Software Library (FSL) to ensure high reproducibility and scalability.

**FIGURE 1:**
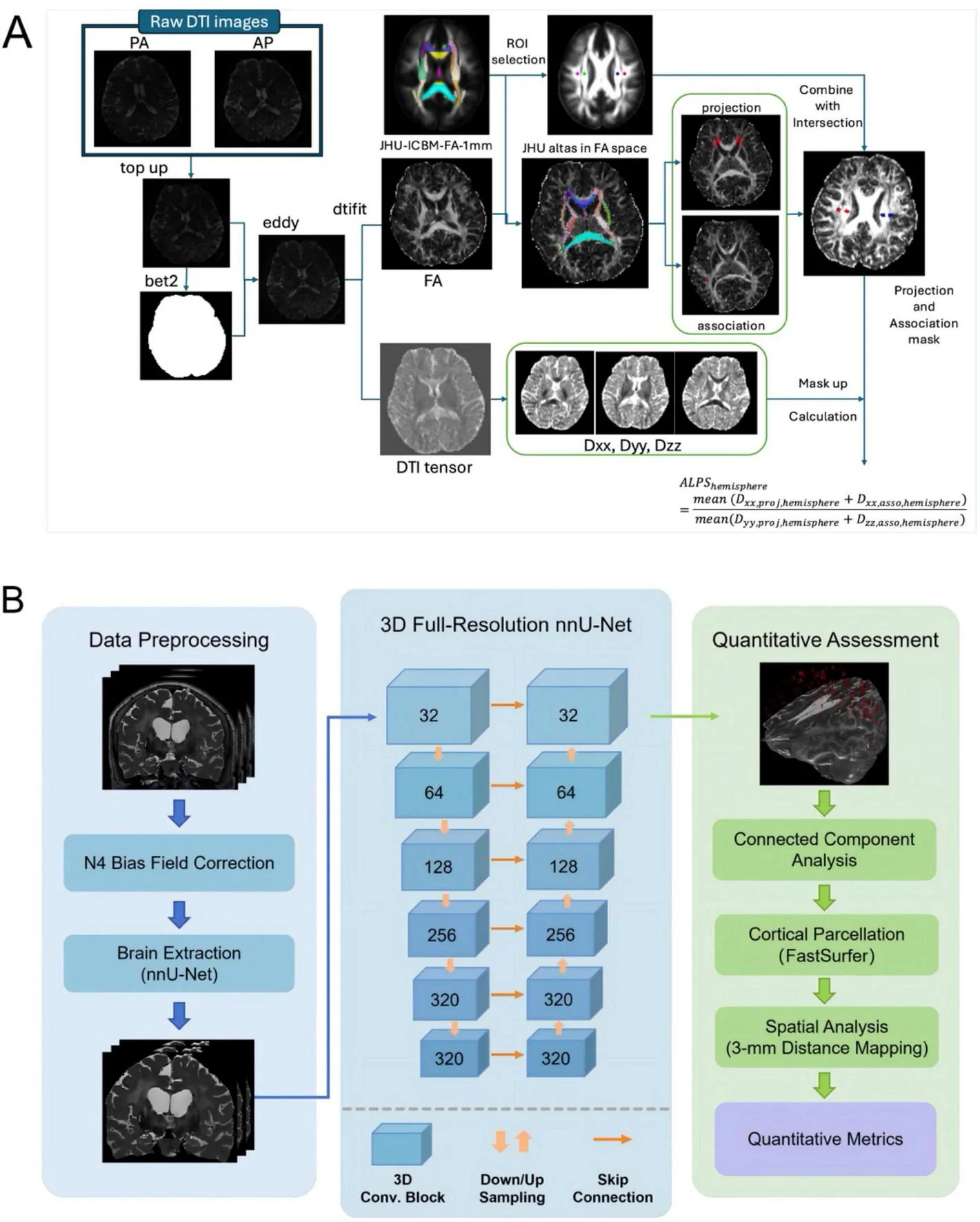
Flow chart for automatic DTI-ALPS index calculation and PVS segmentation. **(A)** The automatic calculation of DTI-ALPS index. The pipeline shows the automatic workflow of DTI-ALPS index acquisition that includes preprocessing with TOPUP and eddy, tensor processing with dtifit, altas-based ROI segmentation and DTI-ALPS calculation. All methods have been implemented in the DTI space to reduce registration loss. **(B)** The automatic segmentation of PVS. All T2WI scans underwent N4 bias field correction and brain extraction. PVS segmentation was performed using the 3D full-resolution configuration of the nnU-Net framework. Connected component analysis and spatial analysis were conducted to quantitatively assess PVS. ALPS, analysis along perivascular space; DTI, diffusion tensor imaging; MRI, magnetic resonance imaging; T2WI, T2-weighted imaging; FA, fractional anisotropy; ROI, region of interest.

Diffusion-weighted data were first processed with a standardized FSL protocol. Parameters of Scanning protocols were automatically read from the JSON sidecar files and written to the acquisition and index files for “topup” and “eddy,” which were used to correct susceptibility distortions, eddy currents, and head motion with identical settings for all participants. Outlier detection and replacement were performed using the –repol option in FSL eddy. After correction, diffusion tensors were estimated in native diffusion space using FSL dtifit, with all b = 0 volumes and diffusion-weighted volumes from both shells (b = 1,000 and 2,000 s/mm^2^) included in the tensor fitting. Fractional anisotropy (FA) and mean diffusivity (MD) maps, as well as the full tensor volumes, were derived from the fitted tensors. FA and MD maps were subsequently processed using tract-based spatial statistics (TBSS). Mean skeletonized FA and MD values were calculated for each participant, and peak width of skeletonized mean diffusivity (PSMD) was additionally derived from the skeletonized MD data. The directional diffusivity maps used for calculation of the DTI-ALPS index were obtained from the same tensor estimates and extracted from the predefined ROIs.

Spatial correspondence across subjects was established by registering each FA image non-linearly to the FMRIB58_FA template. The inverse warp was applied to project the JHU ICBM-DTI-81 white-matter atlas into individual FA space, while the diffusion tensor remained in native space. Therefore, we can derive the projection fibers and association fibers from the desired atlas with labels (23–28 for projection fibers and 41–42 for association fibers). This “atlas-to-subject” strategy enables anatomically consistent region definition without resampling the tensor data (Li W. X. et al., 2024).

To avoid manual region drawing, we used an atlas-based spherical ROIs scheme. In the JHU template space, four small spheres (radius 2.5 mm) were placed at predefined locations within the bilateral superior corona radiata and superior longitudinal fasciculus near the ventricular body. These spheres were warped into each subject’s FA space and intersected with the projection and association masks, yielding tract-specific ROIs in both hemispheres.

Slice selection and hemispheric separation were determined directly from the images. The union of projection and association masks was used to identify the anterior–posterior and inferior–superior extent around the lateral ventricular body. The left–right dimension of the FA image was halved to define the midline, and this midline was used to split the ventricular-level mask into left- and right-sided components, eliminating manual boundary placement.

DTI-ALPS indices were computed from the native-space tensor components. With “fslstats,” we obtained the mean Dxx in projection and association ROIs, and the mean Dyy and mean Dzz in projection and association ROIs, respectively, for each hemisphere. The hemispheric indices were defined as


$${\mathrm{ALPS}}_{\mathrm{hemisphere}}=\frac{\mathrm{mean}\,\left(D_{\mathrm{xx},\mathrm{proj},\mathrm{hemisphere}}+D_{\mathrm{xx},\mathrm{asso},\mathrm{hemisphere}}\right)}{\mathrm{mean}\,\left(D_{\mathrm{yy},\mathrm{proj},\mathrm{hemisphere}}+D_{\mathrm{zz},\mathrm{asso},\mathrm{hemisphere}}\right)}$$

where the overbar denotes the average within each ROI. All steps—from loading sequence parameters through correction, tensor fitting, atlas projection, ROI construction, and index calculation—were executed automatically, providing a standardized, scalable DTI-ALPS framework for neurovascular research.

To identify potential confounding effects of ICH or infarct lesions on DTI-ALPS index calculation, all DTI images were reviewed by an experienced neurologist (YPZ, with 6 years of neuroimaging reading experience). All participants had ROIs located in lesion-free regions except for 2 CAA patients with ICH overlapping ROIs. For cases with lesion involvement, the contralateral DTI-ALPS index was adopted as the final measurement to ensure data reliability.

### 2.5 Quantitative evaluation of PVS

The PVS in the white matter was quantitatively assessed using a deep learning-based framework for accurate, fully automated segmentation from T2WI scans (Figure 1B).

#### 2.5.1 dataset and data preprocessing

The T2WI scans from 14 randomly selected subjects, comprising cases from both CAA and aCSVD, were manually annotated as ground-truth labels for PVS. The dataset was partitioned into training (*n* = 10), validation (*n* = 2), and test (*n* = 2) subsets. Test annotations were produced exclusively via manual delineation to ensure unbiased evaluation.

All T2WI scans underwent N4 bias field correction (Tustison et al., 2010) using ANTs (Avants et al., 2009), followed by an initial brain extraction. As automated skull stripping may introduce inaccuracies, brain masks for nine subjects were manually corrected by an expert and subsequently used to train a dedicated nnU-Net (Isensee et al., 2021) model for brain extraction. This model was applied to the remaining cases to generate the final masks, which exhibited improved accuracy over the initial ANTs-based extraction.

#### 2.5.2 PVS segmentation

Perivascular spaces segmentation was performed using the 3D full-resolution configuration of the nnU-Net framework, which automatically determines optimal preprocessing, network topology, and training hyperparameters based on the dataset characteristics. The network architecture consisted of a six-level 3D U-Net with encoder-decoder symmetry, employing feature widths of 32, 64, 128, 256, 320, and 320 channels across successive resolution levels. The model was trained using a patch size of 128 × 128 × 128 and a batch size of 2.

A composite loss function combining Dice loss (Dice, 1945) and cross-entropy loss with equal weighting was used to balance voxel-wise classification accuracy with overlap-based optimization. Training was performed for 500 epochs using stochastic gradient descent (SGD) with default nnU-Net optimization settings, including learning-rate scheduling and deep supervision across decoder levels. Standard nnU-Net data augmentations were applied during training, including spatial transformations and intensity perturbations.

Model selection was performed on the two-case validation subset, and the weights that achieved the highest validation Dice score were retained as the final model. On the independent two-case test set, the model yielded an average Dice Similarity Coefficient (DSC) of 0.74. The segmented PVS mask was also visually reviewed to avoid obvious deviation and manually corrected if necessary.

#### 2.5.3 Quantitative assessment

Following training, the optimized nnU-Net model was applied to all subjects in the analysis cohort to generate the final PVS segmentation labels. Connected component analysis was used to identify individual PVS, enabling the computation of total burden (count and volume). To investigate the spatial distribution of PVS, a more detailed regional analysis was performed to differentiate between juxtacortical and deep white matter (DWM) burdens. To assess regional distribution, the paired T1WIs were rigidly registered to the T2 space using SimpleITK (Yaniv et al., 2018). Cortical and subcortical parcellations were obtained with FastSurfer (Henschel et al., 2020), and a white-matter distance map was computed to quantify the distance from each voxel to the nearest gray-matter boundary. Voxels located within 3 mm of the gray-matter interface were classified as juxtacortical white matter, whereas more distant voxels were assigned to DWM. Each PVS component was assigned to a region based on the predominant voxel location, and regional PVS metrics [juxtacortical PVS (JPVS) and DWM-PVS] were computed accordingly.

### 2.6 Evaluation of CSVD imaging markers

#### 2.6.1 Evaluation of WMH

White matter hyperintensity was evaluated both visually and automatically. FLAIR images were used to evaluate WMH. Fazekas scale was used for visual rating of WMH, including periventricular WMH (PWMH) and deep WMH (DWMH) (Fazekas et al., 1987). For PWMH: 0 = normal, 1 = small caps or pencil-thin lining, 2 = large caps or smooth halo, and 3 = extended caps or irregular PWM lesions. For DWMH: 0 = normal, 1 = punctate lesions, 2 = beginning of confluence, 3 = confluent DWMH lesions. The total Fazekas score was the summation of DWMH and PWMH scores, ranging from 0 to 6. Furthermore, WMH volume was automatically segmented using the lesion prediction algorithm (Schmidt, 2017) as implemented in the lesion segmentation tool toolbox^1^ for Statistical Parametric Mapping 12 (SPM 12)^2^. The segmentations were visually checked and manually corrected if necessary.

#### 2.6.2 Evaluation of brain atrophy

Structural T1WI data were processed using FreeSurfer version 8.0.0.^3^ The standard “recon-all” pipeline was employed, including motion correction, removal of non-brain tissue, automated Talairach transformation, intensity normalization, segmentation of subcortical white matter and deep gray matter structures, tessellation of the gray and white matter boundary, automated topology correction, and surface deformation to optimally place the white and pial surfaces. All segmentations and reconstructed surfaces underwent rigorous visual quality control and bias correction, as described in detail in our previous research (Liu et al., 2023). Brain atrophy was evaluated using the brain parenchymal fraction (BPF), defined as the proportion of brain tissue volume to total intracranial volume (Pan et al., 2026). The brain tissue volume aggregated that of the white and gray matter.

#### 2.6.3 Evaluation of other CSVD imaging markers

Other CSVD imaging markers, including basal ganglia (BG) -PVS, lacunes, CMBs, and cortical superficial siderosis (cSS), were also evaluated visually by experienced neurologists according to the Standards for Reporting Vascular Changes in Neuroimaging (STRIVE) criteria (Duering et al., 2023; Wardlaw et al., 2013), with assessments blinded to clinical information. BG-PVS were rated on axial T2WI using an established visual rating scale (Potter et al., 2015): 0 = no PVS, 1 = 1–10 PVS, 2 = 11–20 PVS, 3 = 21–40 PVS, and 4 = >40 PVS. The other markers were recorded as present or absent. The intra- and inter-rater Cohen’s κ for lacunes, CMBs, BG-PVS, and cSS ranged from 0.818 to 1.00, indicating good reliability.

Based on prior evaluations, the total CSVD score was also rated according to Wardlaw’s standard (Staals et al., 2014). The CSVD score ranges from 0 to 4, with 1 point for each of the following: WMH burden (PWMH Fazekas ≥ 3 or DWMH Fazekas ≥ 2), the presence of lacunes, the presence of CMBs, and BG-PVS > 10.

### 2.7 Statistical analysis

Continuous data were described as mean ± standard deviation (SD) or as median with interquartile range for normally and non-normally distributed data, respectively. Categorical variables were described as frequencies with percentages. Comparisons between groups were conducted using Student’s *t*-test (or Mann-Whitney test), Kruskal-Wallis test, and the Chi-square (χ^2^) test (or Fisher’s exact test) for continuous and categorical variables, as appropriate. Spearman’s correlation analyses were performed to explore associations between DTI-ALPS index and quantified PVS burden. Moreover, general linear regression models were performed to explore potential clinical and imaging factors associated with DTI-ALPS index, adjusting for age and sex. The Benjamini–Hochberg false discovery rate (FDR) procedure was applied to adjust *p*-values for multiple comparisons across the primary predictors. Both nominal and FDR-adjusted *p*-values were reported. All these analyses were conducted in both the whole cohort and subgroups of CAA and aCSVD. For cognitive outcomes, MoCA scores were analyzed using general linear regression, whereas MMSE scores, which showed a left-skewed distribution, were transformed to the open interval (0, 1) and analyzed using beta regression with a logit link and robust standard errors. Since MoCA scores had been education-corrected according to the standard scoring procedure (+1 point for participants with ≤12 years of education) during data collection, MoCA models were adjusted for age and sex, whereas MMSE models were adjusted for age, sex, and education.

All statistical analyses were conducted using Stata (version 18.0, StataCorp LLC) and SPSS (version 30.0, IBM Corp). A two-tailed *p* < 0.05 was considered statistically significant.

## 3 Results

### 3.1 Demographic, clinical, and imaging characteristics

A total of 134 participants (56 probable CAA and 78 aCSVD) were finally included in the analyses, with a mean age of 64.9 ± 10.0 years and 64.9% male. The demographic, clinical, and imaging characteristics of the participants were summarized in Table 1. The CAA patients were older than aCSVD patients and presented with less hypertension. The two groups were comparable in other vascular risk factors, including diabetes, hyperlipidemia, smoking, and alcohol drinking. Moreover, cognitive performance was also comparable between the two groups.

**TABLE 1:** Characteristics of participants.

Variables	Total (*n* = 134)	CAA (*n* = 56)	aCSVD (*n* = 78)	*P*-value
Age-y	64.9 ± 10.0	67.2 ± 9.4	63.3 ± 10.2	0.025
Sex (male, %)	87 (64.9)	36 (64.3)	51 (65.4)	0.895
Hypertension (%)	102 (76.1)	34 (60.7)	68 (87.2)	<0.001
Diabetes (%)	23 (17.2)	9 (16.1)	14 (18.0)	0.776
Hyperlipidemia (%)	54 (40.3)	23 (41.1)	31 (39.7)	0.877
Smoking (%)	54 (40.3)	23 (41.1)	31 (39.7)	0.877
Drinking alcohol (%)	51 (38.1)	20 (35.7)	31 (39.7)	0.636
Education (high school or above, %)	71 (53.0)	29 (51.8)	42 (53.9)	0.814
MoCA*	18 ± 5	19 ± 5	18 ± 5	0.660
MMSE*	26 (23–28)	26 (24–28)	27 (22–28)	0.933
ICH presence (%)	37 (27.6)	23 (41.1)	10 (18.0)	0.003
Lobar ICH (%)	23 (17.2)	23 (41.1)	0 (0)	<0.001
CMBs presence (%)	116 (86.6)	49 (87.5)	67 (85.9)	0.788
cSS presence (%)	26 (19.4)	26 (19.4)	0 (0)	<0.001
BG-PVS grades	3 (2–4)	3 (2–3)	3 (2–4)	0.005
Lacune presence (%)	102 (76.1)	30 (53.6)	72 (92.3)	<0.001
Fazekas scores				
PWMH	2 (2–3)	2 (1–2)	2 (2–3)	<0.001
DWMH	2 (2–3)	2 (2–3)	3 (2–3)	0.041
Total	5 (4–6)	4 (3–5)	5 (4–6)	<0.001
CSVD score	4 (3–4)	3 (3–4)	4 (4–4)	<0.001
WMH volume	16.3 (9.9–27.3)	11.0 (7.0–22.0)	20.8 (12.3–31.2)	<0.001
FA	0.43 (0.39–0.45)	0.44 (0.41–0.46)	0.41 (0.38–0.45)	0.003
MD, ×10^–3^	0.67 (0.63–0.71)	0.66 (0.63–0.70)	0.68 (0.64–0.72)	0.042
PSMD,×10^–3^	0.46 (0.36–0.60)	0.46 (0.36–0.60)	0.50 (0.37–0.62)	0.034
BPF^#^	0.67 ± 0.03	0.66 ± 0.04	0.66 ± 0.03	0.363

*Available for 114 participants. #Two missing data, available for 132 participants. aCSVD, arteriolosclerosis cerebral small vessel disease; BG, basal ganglia; BPF, brain parenchymal fraction; CAA, cerebral amyloid angiopathy; CMB: cerebral microbleed; CSVD, cerebral small vessel disease; cSS, cortical superficial siderosis; DWMH, deep white matter hyperintensity; FA, fractional anisotropy; ICH, intracerebral hemorrhage; MD, mean diffusivity; MoCA, Montreal Cognitive Assessment; MMSE, Mini-Mental State Examination; PSMD, peak width of skeletonized mean diffusivity; PVS, perivascular space; PWMH, periventricular white matter hyperintensity; WMH, white matter hyperintensity.

Compared with CAA patients, aCSVD patients had more severe BG-PVS and WMH, a higher proportion of lacunes, and higher total CSVD scores (all *p* < 0.05).

### 3.2 Disease-specific differences in the DTI-ALPS index and quantified PVS

Compared with the CAA group, aCSVD patients showed significantly lower DTI-ALPS index values (mean ± SD: 1.12 ± 0.13 vs. 1.23 ± 0.13, *p* < 0.001, Figure 2 and Supplementary Table 2). However, quantitative measures of PVS burden were significantly higher in CAA than in aCSVD (all *p* < 0.05, Figure 2 and Supplementary Table 2).

**FIGURE 2:**
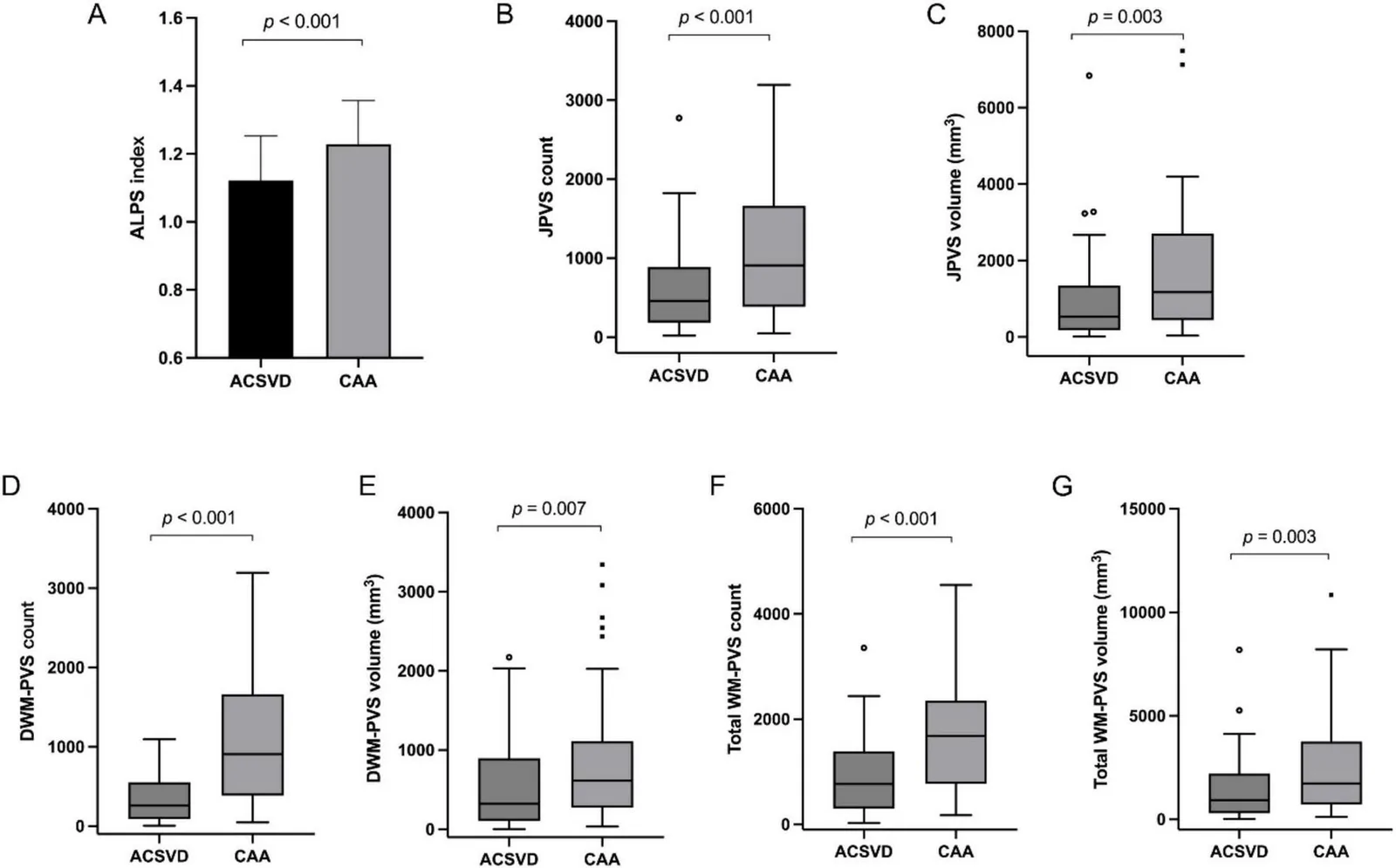
Disease-specific differences of the diffusion tensor imaging (DTI)-ALPS index and quantified PVS. **(A)** Bar plot showing significantly higher DTI-ALPS index in the CAA group compared with the aCSVD group (*p* < 0.001). **(B–G)** Box plots demonstrating significantly greater JPVS count [**(B)**, *p* < 0.001], JPVS volume [**(C)**, *p* = 0.003], DWM-PVS count [**(D)**, *p* < 0.001], DWM-PVS volume [**(E)**, *p* = 0.007], total WM-PVS count [**(F)**, *p* < 0.001], and total WM-PVS volume [**(G)**, *p* = 0.003] in the CAA group versus the aCSVD group. aCSVD, arteriolosclerosis cerebral small vessel disease; ALPS, diffusion tensor image analysis along the perivascular space; CAA, cerebral amyloid angiopathy; JPVS, juxtacortical perivascular spaces; DWM-PVS, deep white matter perivascular spaces; WM-PVS, white matter perivascular spaces.

### 3.3 Disease-specific differences in the association between the DTI-ALPS index and clinical factors

The correlation between the DTI-ALPS index and clinical factors is summarized in Table 2 and Supplementary Table 3. In the univariable analyses, age, sex, and other clinical factors were examined individually for their associations with the DTI-ALPS index. Female sex (β = 0.10, *p* = 0.001) was associated with a lower DTI-ALPS index in the aCSVD group, whereas this association did not reach statistical significance in the CAA group. In the multivariable analyses, the other clinical factors were examined separately with adjustment for age and sex. However, after FDR correction, none reached statistical significance.

**TABLE 2:** Correlations between DTI-ALPS index and clinical factors*.

Clinical factors	Total (*n* = 134)			aCSVD (*n* = 78)			CAA (*n* = 56)		
	β (95% CI)	*P*-value	FDR-adjusted *p*-value	β (95% CI)	*P*-value	FDR-adjusted *p*-value	β (95% CI)	*P*-value	FDR-adjusted *p*-value
Hypertension	−0.06 (−0.12, −0.01)	0.020	0.105	−0.01 (−0.09, 0.07)	0.797	0.797	−0.03 (−0.11, 0.04)	0.339	0.665
Diabetes	0.03 (−0.03, 0.09)	0.323	0.388	0.08 (0.02, 0.16)	0.014	0.084	−0.05 (−0.14, 0.04)	0.277	0.665
Hyperlipidemia	0.03 (−0.02, 0.08)	0.262	0.388	0.03 (−0.03, 0.10)	0.270	0.405	0.03 (−0.04, 0.10)	0.443	0.665
Smoke	−0.03 (−0.09, 0.03)	0.300	0.388	−0.06 (−0.12, 0.01)	0.117	0.234	−0.01 (−0.10, 0.08)	0.762	0.860
Drink	−0.06 (−0.11, −0.004)	0.035	0.105	−0.06 (−0.12, 0.01)	0.090	0.234	−0.05 (−0.13, 0.03)	0.209	0.665
Education	0.01 (−0.04, 0.05)	0.748	0.748	0.01 (−0.04, 0.07)	0.676	0.797	0.01 (−0.06, 0.07)	0.860	0.860

*General linear regression models were fitted with the DTI-ALPS index as the dependent variable. Each clinical marker was examined separately as the independent variable, with adjustment for age and sex. aCSVD, arteriolosclerosis cerebral small vessel disease; DTI-ALPS, diffusion tensor imaging analysis along perivascular space; CAA, cerebral amyloid angiopathy; CI, confidence interval; FDR, false discovery rate.

### 3.4 Disease-specific differences in the association between DTI-ALPS index and imaging factors

The correlation between the DTI-ALPS index and imaging factors is summarized in Table 3 and Supplementary Table 4. In both the aCSVD and CAA populations, the association between DTI-ALPS index and WMH volume, MD, FA, and PSMD was consistently significant (all *p* < 0.05), even after adjusting for age and sex, and FDR adjustment. However, the association between DTI-ALPS index and established CAA imaging markers (including lobar ICH, strictly lobar CMBs, and cSS) did not reach statistical significance.

**TABLE 3:** Correlations between DTI-ALPS index and imaging factors*.

Imaging factors	Total (*n* = 134)			aCSVD (*n* = 78)			CAA (*n* = 56)		
	β (95% CI)	*P*-value	FDR-adjusted *p*-value	β (95% CI)	*P*-value	FDR-adjusted *p*-value	β (95% CI)	*P*-value	FDR-adjusted *p-*value
ICH presence	0.01 (−0.05, 0.06)	0.815	0.869	0.002 (−0.07, 0.08)	0.938	0.938	0.06 (−0.13, 0.004)	0.066	0.146
Lobar ICH	0.04 (−0.02, 0.10)	0.169	0.193	-	-		0.06 (−0.13, 0.004)	0.066	0.146
CMBs presence	−0.05 (−0.11, 0.020)	0.169	0.193	−0.07 (−0.15, 0.01)	0.073	0.105	−0.02 (−0.12, 0.08)	0.695	0.695
Strictly lobar CMBs	0.09 (0.04, 0.14)	**0.001**	**0.001**	-	-		0.02 (−0.05, 0.09)	0.528	0.563
cSS presence	0.06 (0.01, 0.12)	**0.030**	**0.040**	–	–	–	−0.02 (−0.09, 0.05)	0.518	0.563
BG-PVS grades	0.002 (−0.03, 0.03)	0.902	0.902	0.02 (0.02)	0.214	0.232	0.03 (−0.01, 0.08)	0.158	0.230
Lacune presence	−0.10 (−0.15, −0.05)	**<0.001**	**<0.001**	−0.08 (−0.18, 0.03)	0.146	0.172	−0.06 (−0.13, 0.01)	0.078	0.146
Fazekas scores									
PWMH	−0.08 (−0.11, −0.05)	**<0.001**	**<0.001**	−0.08 (−0.12, −0.02)	**0.001**	**0.002**	−0.04 (−0.09, 0.01)	0.093	0.149
DWMH	−0.06 (−0.09, −0.03)	**<0.001**	**<0.001**	−0.05 (−0.09, −0.02)	**0.006**	**0.011**	−0.03 (−0.07, 0.02)	0.215	0.265
Total	−0.04 (−0.06, −0.03)	**<0.001**	**<0.001**	−0.04 (−0.06, −0.02)	**0.001**	**0.002**	−0.02 (−0.05, 0.003)	0.082	0.146
WMH volume^#^	−0.07 (−0.09, −0.05)	**<0.001**	**<0.001**	−0.05 (−0.07, −0.03)	**0.001**	**0.002**	−0.08 (−0.12, −0.04)	**0.002**	**0.006**
CSVD score	−0.05 (−0.07, −0.02)	**0.001**	**0.001**	−0.03 (−0.06, 0.004)	0.083	0.108	−0.03 (−0.07, 0.01)	0.205	0.265
BPF	1.58 (0.87, 2.29)	**<0.001**	**<0.001**	0.94 (0.04, 1.84)	**0.044**	0.072	1.80 (0.74, 2.86)	**0.002**	**0.006**
FA	1.58 (1.11, 2.05)	**<0.001**	**<0.001**	1.17 (0.59, −1.76)	**<0.001**	**0.002**	1.63 (0.86, −2.40)	**<0.001**	**0.003**
MD, ×10^–3^	−0.91 (−1.22, −0.59)	**<0.001**	**<0.001**	−0.89 (−1.34, −0.43)	**<0.001**	**0.002**	−0.74 (−1.13, −0.35)	**<0.001**	**0.003**
PSMD, ×10^–3^	−0.36 (−0.47, −0.24)	**<0.001**	**<0.001**	−0.36 (−0.52, −0.20)	**<0.001**	**0.002**	−0.30 (−0.44, −0.16)	**<0.001**	**0.003**

*General linear regression models were fitted with the DTI-ALPS index as the dependent variable. Each imaging marker was examined separately as the independent variable, with adjustment for age and sex. ^#^Standardized transformed (Z-score). aCSVD, arteriolosclerosis cerebral small vessel disease, DTI-ALPS, diffusion tensor imaging analysis along perivascular space, BG, basal ganglia, BPF, brain parenchymal fraction, CAA, cerebral amyloid angiopathy, CI, confidence interval, CMB, cerebral microbleed, CSVD, cerebral small vessel disease, cSS, cortical superficial siderosis, DWMH, deep white matter hyperintensity; FDR, false discovery rate; ICH, intracerebral hemorrhage; PVS, perivascular space; PWMH, periventricular white matter hyperintensity; PSMD, peak width of skeletonized mean diffusivity; WMH, white matter hyperintensity. Bold values indicate statistical significance (*P* < 0.05).

### 3.5 Association between the DTI-ALPS index and PVS

Of note, the associations between DTI-ALPS index and PVS metrics, including BG-PVS grades, JPVS count and volume, DWM-PVS count and volume, and total white matter PVS count and volume, were not statistically significant in either the whole cohort or in the subgroups (Table 4).

**TABLE 4:** Correlations between DTI-ALPS index and PVS burden.

PVS metrics	Total (*n* = 134)	CAA (*n* = 56)	aCSVD (*n* = 78)
JPVS count	0.13 (0.125)	−0.01 (0.939)	0.07 (0.549)
DWM-PVS count	0.16 (0.071)	0.10 (0.456)	0.04 (0.709)
Total WM-PVS count	0.16 (0.072)	0.03 (0.848)	0.05 (0.673)
JPVS volume	0.06 (0.500)	−0.15 (0.274)	0.06 (0.577)
DWM-PVS volume	0.05 (0.539)	−0.10 (0.468)	0.03 (0.811)
Total WM-PVS volume	0.05 (0.544)	−0.13 (0.356)	0.04 (0.731)
BG-PVS grade	−0.03 (0.769)	0.07 (0.606)	0.06 (0.586)

The data were expressed as Spearman’s ρ (*p*-value). aCSVD, arteriolosclerosis cerebral small vessel disease; DTI-ALPS, diffusion tensor imaging analysis along perivascular space; CAA, cerebral amyloid angiopathy; BG, basal ganglia; DWM, deep white matter; JPVS, juxtacortical perivascular space; PVS, perivascular space; WM, white matter.

### 3.6 Association between the DTI-ALPS index and cognition

Cognitive data were available for 114 participants (Table 5). Among these patients, higher DTI-ALPS index values were significantly associated with better global cognition on both the MMSE (β = 1.74, *p* = 0.003) and the MoCA (β = 12.73, *p* < 0.001). After stratification by CSVD subtype, the association remained robust in the aCSVD group (MMSE: β = 2.00, *p* = 0.006; MoCA: β = 17.57, *p* < 0.001). However, the association did not reach statistical significance in the CAA group.

**TABLE 5:** Association between DTI-ALPS and cognitive function*.

Population	Unadjusted		Adjusted^#^	
	Coefficient (95% CI)	*P*-value	Coefficient (95% CI)	*P*-value
Whole cohort (n = 114)				
MMSE	1.65 (0.44, 2.86)	0.007	1.74 (0.60, 2.88)	0.003
MoCA	11.23 (4.72, 17.73)	0.001	12.73 (6.11, 19.35)	<0.001
CAA (n = 47)				
MMSE	2.12 (−0.05, 4.29)	0.056	2.12 (−0.22, 4.45)	0.076
MoCA	7.66 (−3.83, 19.16)	0.186	10.47 (−1.01, 21.94)	0.073
aCSVD (n = 67)				
MMSE	1.91 (0.54, 3.28)	0.006	2.00 (0.57, 3.43)	0.006
MoCA	16.62 (7.77, 25.48)	<0.001	17.57 (8.03, 27.10)	<0.001

*Linear regression for MoCA and beta regression with a logit link for MMSE were performed with the DTI-ALPS index as the independent variable. #MoCA models were adjusted for age and sex, whereas MMSE models were adjusted for age, sex, and years of education. aCSVD, arteriolosclerosis cerebral small vessel disease; CAA, cerebral amyloid angiopathy; CI, confidence interval; DTI-ALPS, diffusion tensor imaging analysis along perivascular space; MoCA, Montreal Cognitive Assessment; MMSE, Mini-Mental State Examination.

## 4 Discussion

In the present ultra-high-field MRI study, we systematically evaluated DTI-ALPS index performance across two major CSVD subtypes with distinct vascular pathologies. The findings indicated that the DTI-ALPS index varies significantly between aCSVD and CAA, with the index in aCSVD being significantly lower than that in CAA. However, across both groups, DTI-ALPS index was consistently associated with WMH burden and white matter microstructural measures, including skeletonized FA, skeletonized MD, and PSMD. In contrast, associations of the DTI-ALPS index with PVS burden in either group and with established CAA imaging markers did not reach statistical significance. Collectively, our findings provide disease-specific evidence that DTI-ALPS index performance is strongly dependent on disease context but consistently associated with white matter microstructural integrity across distinct angiopathies. These results may contribute to a better understanding of the DTI-ALPS index characteristics underlying distinct pathologies and may refine the interpretation of the DTI-ALPS index.

### 4.1 The different characteristics and mechanisms of DTI-ALPS index in CAA and aCSVD

In this study, we evaluated DTI-ALPS index alterations in CAA and aCSVD, which represent distinct pathological subtypes of CSVD. Previous studies have reported significantly lower DTI-ALPS indices in both CAA and aCSVD compared with age-matched healthy controls (Huang et al., 2025; Xu et al., 2022). However, direct comparisons of the DTI-ALPS index between CAA and aCSVD remain scarce. Although healthy controls were unavailable in the present study, the DTI-ALPS values observed in our CAA and aCSVD groups were consistent with those reported in previous studies (Huang et al., 2025; Xu et al., 2022). Notably, our results showed a significant lower DTI-ALPS index in aCSVD than in CAA. This discrepancy aligned with the pathological differences underlying CAA and aCSVD. Although both conditions are associated with impaired glymphatic clearance, the underlying mechanisms differ substantially. The pathological damage of CAA primarily involves the cortex and leptomeninges, characterized by abnormal deposition of Aβ in the vessel walls, which directly disrupts perivascular drainage pathways and leads to impaired interstitial fluid clearance (Hawkes et al., 2014; Koemans et al., 2023; Pantoni, 2010). In contrast, aCSVD involves arteriolosclerosis of deep perforating vessels, resulting in reduced arterial pulsatility, chronic hypoperfusion, secondary white matter damage, and reduced convective perivascular flow (Benveniste and Nedergaard, 2022; Cuadrado-Godia et al., 2018; Mestre et al., 2017; Pantoni, 2010). The supposed anatomical rationale of DTI-ALPS index is based on the approximately orthogonal arrangement of the presumed PVS and major white-matter fiber tracts in the periventricular white matter, allowing diffusivity in the direction approximately parallel to the PVS to be evaluated relative to diffusivity in directions perpendicular to the PVS (Taoka et al., 2017). The ROIs where the DTI-ALPS index is measured, namely the corona radiata, are not typically involved in CAA, but rather are typically affected by aCSVD pathology. Moreover, the GS relies on PVS as a pathway for cerebrospinal and interstitial fluid exchange, which works closely linked to the anatomical distribution of vessels and their drainage pathways. The DTI-ALPS index reflects relative diffusivity along the presumed PVS direction around the deep medullary veins (DMV) compared with diffusivity in orthogonal directions (Taoka et al., 2017). The DMV primarily drain into the subependymal and intracerebral veins, largely independent of the superficial venous network (Huang and Wolf, 1964). Meanwhile, the cortical vessels that penetrate from the superficial to the deep white matter are relatively few (Duvernoy et al., 1981). Therefore, DTI-ALPS index may have limited sensitivity for detecting glymphatic clearance along cortical vessels affected by CAA pathology. Supportively, a recent study compared the DTI-ALPS index with glymphatic MRI and observed a limited correlation between DTI-ALPS index and glymphatic functional metrics measured in cortical regions (Mossige et al., 2026). Consequently, the DTI-ALPS index may be unable to capture glymphatic dysfunction associated with CAA.

Moreover, vascular risk factors also explained great variability in DTI-ALPS index across CAA and aCSVD. While demographic and vascular risk factors have consistently been shown to be associated with reduced DTI-ALPS index (Benveniste and Nedergaard, 2022; Chen et al., 2025; Li W. X. et al., 2024; Zhang Y. et al., 2021), these factors are also major risk factors for aCSVD (Benveniste and Nedergaard, 2022; Cuadrado-Godia et al., 2018; Furuta et al., 1991; Pantoni, 2010). Our results consistently supported the association of female sex with reduced DTI-ALPS index, primarily in aCSVD patients. However, such associations were not statistically significant in CAA. This discrepancy was also consistent with the underlying angiopathic mechanisms, as CAA was predominantly driven by Aβ accumulation rather than by vascular risk factors (Hawkes et al., 2014; Xu et al., 2023).

### 4.2 The DTI-ALPS index is consistently associated with white matter damage but not with PVS

Despite the significant heterogeneity of DTI-ALPS index across subgroups, the associations between DTI-ALPS index and WMH volume and white matter microstructural integrity (indicated by FA, MD, and PSMD) were consistently significant across CAA and aCSVD. These results were consistent with previous studies (Lin et al., 2025; Xu et al., 2022; Zhao et al., 2025), supporting the confounding effects of white matter damage and brain structure changes on DTI-ALPS index.

On the contrary, no statistically significant associations between DTI-ALPS and PVS burden was observed in our study. Although both PVS and DTI-ALPS index were regarded as indicators of glymphatic function, the association between DTI-ALPS index and PVS was inconsistent across previous research (Xu et al., 2022; Zhang W. et al., 2021). Our present study employed an automated method to carefully quantify the number and volume of PVS in white matter and analyzed the association between DTI-ALPS index and PVS burden. The ultra-high-field 5.0T MRI was applied to enable more accurate PVS segmentation than conventional MRI (Liu et al., 2025). However, associations between the DTI-ALPS index and quantitative white matter PVS measures did not reach statistical significance in either CAA or aCSVD. Neither global nor sectional analyses (JPVS and DWM-PVS) showed statistically significant association between PVS and DTI-ALPS index. Moreover, the association between BG-PVS and DTI-ALPS index was also not significant. These results indicate that PVS and DTI-ALPS index may be independent of each other and convey distinct information. Given the complexity of GS, the DTI-ALPS index may be unable to capture the full extent of glymphatic function.

### 4.3 Limited association between DTI-ALPS index and CAA

Research focused on the association between DTI-ALPS index and CAA was limited. Several previous studies reported reduced DTI-ALPS index in CAA compared with healthy controls (Lin et al., 2025; Xu et al., 2022; Zhao et al., 2025). However, Lin et al. (2025) indicated that although DTI-ALPS index was lower in CAA-related ICH patients than in healthy controls, the group differences were not significant after adjusting for CSVD burden, suggesting that the DTI-ALPS index reduction is more likely attributable to CSVD severity than to CAA-related ICH. Although healthy controls were unavailable in our present research, the DTI-ALPS value in CAA patients was comparable to that in previous research (Lin et al., 2025; Xu et al., 2022), supporting the reliability of our results. However, in both the present research and previous studies (Lin et al., 2025; Xu et al., 2022), associations between DTI-ALPS index and established CAA imaging markers, including lobar CMBs, cSS, and increased PVS in white matter (including centrum semiovale PVS), did not reach statistical significance. Moreover, the correlation between reduced DTI-ALPS index and Aβ burden in CAA was inconclusive (Satpathi et al., 2025; Zhao et al., 2025). The poor correlation between established CAA imaging markers and DTI-ALPS index further suggests that DTI-ALPS index may be a poor surrogate for GS function in CAA.

The correlation between DTI-ALPS index and cognition was statistically significant in aCSVD but not in CAA. Nonetheless, our results support an overall association between DTI-ALPS index and cognitive performance in the combined CSVD cohort, consistent with previous studies (Hein et al., 2025; Li H. et al., 2024; Wang et al., 2023; Xu et al., 2022; Zhang W. et al., 2021). Previous studies have revealed that lower DTI-ALPS index is associated with both cross-sectional poor cognitive performance and longitudinal cognitive decline in CSVD (Li H. et al., 2024; Wang et al., 2023). While cross-sectional studies found that lower DTI-ALPS index was associated with poorer memory, executive function, visual-spatial, and language performance (Hong et al., 2024), longitudinal studies observed a significant association with global cognition and processing speed over time (Li H. et al., 2024). Amyloid burden, brain atrophy, and WMH iron burden could be mediators between DTI-ALPS index and cognition (Huang et al., 2024; Qiu et al., 2025). Despite consistent associations between DTI-ALPS index and cognition across previous studies, the CSVD patients in these studies (Hein et al., 2025; Li H. et al., 2024; Wang et al., 2023; Zhang W. et al., 2021) were enrolled based on imaging markers and thus represented a mixed CSVD population without differentiating specific etiologies. Research focused on CAA was limited and showed an inconsistent relationship between DTI-ALPS index and cognition (Lin et al., 2025; Xu et al., 2022; Zhao et al., 2025). Xu et al. (2022) reported that DTI-ALPS index was positively correlated with global cognition (MMSE and MoCA) after adjusting for age and sex. Lin et al. (2025) subsequently observed significant associations between DTI-ALPS index and language, executive control, and processing speed in patients with CAA-related hemorrhage. By contrast, Zhao et al. (2025) found no relationship between DTI-ALPS index and cognitive performance on the MMSE, MoCA, or Clinical Dementia Rating scale. In the present study, the association between DTI-ALPS index and global cognition was not significant when the analysis was restricted to the CAA subgroup. Given the limited sample sizes in both the current and previous studies, a large-scale study focused on CAA is warranted to further elucidate the relationship between DTI-ALPS index and cognitive function in this specific population.

### 4.4 Caution for interpretation of the DTI-ALPS index

Although DTI-ALPS index has been widely used as an indicator for evaluating glymphatic function, the interpretation of the DTI-ALPS index should still be done with caution. On the one hand, the ROI for DTI-ALPS calculation was small and restricted. Therefore, the DTI-ALPS index calculation is largely influenced by localized vascular pathologies and thus varies across angiopathies with heterogeneous vascular involvement. Moreover, the DTI-ALPS index may be unable to reflect the entire GS function (Taoka et al., 2024). As a complex system, the complete functions and pathways of the GS remain unclear. Glymphatic clearance through CSF-ISF exchange in the deep white matter, which is the ROI used to calculate DTI-ALPS index, plays only a partial role in GS function (Ringstad et al., 2018; Ringstad, 2024). The decoupling of DTI-ALPS index from PVS, a traditional indicator of glymphatic clearance, further highlighted the complexity of GS. On the other hand, the calculation of the DTI-ALPS index was influenced by changes in white matter microstructure and axonal geometry (Schilling et al., 2025). The DTI-ALPS index assessed the Brownian motion of water molecules in the radial direction at the level of the lateral ventricular body, which was assumed to be parallel to the medullary veins and to reflect glymphatic flow (Taoka et al., 2017). The calculation relies on the assumption of radial symmetry in the diffusion within and around white matter tracts, enabling post-processing isolation of PVS-specific diffusivity fields (Taoka et al., 2017). However, recent studies have revealed that radial asymmetry is present in the ROIs of DTI-ALPS index as well as in widespread brain structures (Schilling et al., 2025; Wright et al., 2024). Therefore, the calculation of DTI-ALPS index reflects not only perivascular diffusivity but also the axonal contributions (Schilling et al., 2025; Wright et al., 2024). Moreover, the orientation of medullary veins was substantially heterogeneous, which challenges the assumption that PVS is consistently perpendicular to the white matter tracts (Schilling et al., 2025). Furthermore, by directly comparing DTI-ALPS index with glymphatic MRI, Mossige et al. (2026) revealed a limited association between DTI-ALPS index and tracer dynamics, with sparse tracer enhancement within the ROIs of DTI-ALPS index calculation. Therefore, the authors (Mossige et al., 2026) also pointed out that the DTI-ALPS index may reflect local white matter integrity rather than glymphatic clearance. Consistent with previous studies, our research also supported the correlation between DTI-ALPS index and white matter damage. Collectively, DTI-ALPS index is more likely to reflect tissue integrity than universal glymphatic function. The interpretation of DTI-ALPS index as a surrogate for glymphatic function should still be approached with caution. The clinical significance of DTI-ALPS index still awaits further confirmation by more pathologically related studies.

### 4.5 Strengths and limitations

A main strength of our study is the direct and detailed comparative analysis of DTI-ALPS index across two CSVD subtypes with distinct spatial patterns of vascular damage. Moreover, the 5.0T MRI facilitated high-spatial-resolution imaging and detailed visualization of small anatomical structures, such as PVS (Liu et al., 2025). The study still has several limitations. First, the present study was single-center research with a relatively small sample size. Multicenter studies with large sample sizes are needed to verify the findings. Second, patients were enrolled based on clinical and neuroimaging findings. In the present study, aCSVD was primarily identified by patterns of ICH and CMBs, as deep ICH and mixed and deep CMBs were predominantly associated with aCSVD pathology (Sheth, 2022; Ii et al., 2020; Pantoni, 2010). However, because there is no established clinical diagnostic criterion or consensus for aCSVD, diagnostic criteria may vary across studies, potentially introducing bias. Meanwhile, including patients with mixed CMBs, who may harbor both aCSVD and CAA pathologies, could attenuate the between-group distinction. Nevertheless, despite this potential attenuation, the differences in the DTI-ALPS index between the two groups remained significant. Therefore, it may be reasonable to postulate that a significant difference in the DTI-ALPS index persists between patients with pure CAA and those with pure aCSVD. Future studies incorporating pathological validation will provide more rigorous evidence. Third, the absence of an age-matched healthy control group precluded determination of the absolute degree of DTI-ALPS abnormality in either CAA or aCSVD in the present study. Therefore, the clinical utility and disease-specific interpretation of DTI-ALPS, particularly in CAA, requires further validation against age-matched healthy controls. Additionally, due to the cross-sectional design, the causal relationships among DTI-ALPS index, CSVD markers, and cognitive function remain unclear. Longitudinal studies are warranted to clarify these potential causal relationships. Furthermore, the use of 5.0T MRI in the present study may limit the generalizability and direct comparability of our findings with previous 3.0T DTI-ALPS studies. Future cross-field-strength validation is warranted. Moreover, the diffusion tensors were estimated based on multi-shell data which may limited the direct comparisons of absolute diffusion measures with studies using conventional single-shell DTI. Therefore, cross-study comparisons of absolute FA, MD, and DTI-ALPS values should be interpreted with caution. Additionally, vascular risk factors such as smoking and alcohol use were analyzed as categorical variables because quantitative exposure data were unavailable. Therefore, potential dose–response effects could not be assessed. Finally, the relatively small sample sizes may have limited the statistical power, particularly for subgroup analyses. Accordingly, these subgroup-specific findings should be regarded as exploratory rather than confirmatory, interpreted with caution, and validated in larger, adequately powered cohorts.

## 5 Conclusion

Our study systematically characterized DTI-ALPS index alterations across two CSVD phenotypes with distinct spatial patterns and mechanisms of vascular injury. The results suggested that the DTI-ALPS index varied significantly across different angiopathies but was consistently and significantly associated with white matter microstructural injury across different disease groups. However, associations with established CAA imaging markers did not reach statistical significance, and the disease-specific interpretation of DTI-ALPS in CAA remains uncertain. Moreover, the association between DTI-ALPS index and PVS burden did not reach statistical significance in either group. These findings underscore the importance of considering disease context and structural determinants in the clinical application of DTI-ALPS index and may refine the interpretation of DTI-ALPS index. Our findings further emphasize that the interpretation of DTI-ALPS index as a surrogate for glymphatic function should be done with caution. Further studies are warranted to determine the biological value of DTI-ALPS index and the applicability across different disease entities.

## Data Availability

The raw data supporting the conclusions of this article will be made available by the authors, without undue reservation.

## Footnotes

1
https://www.statistical-modeling.de/lst.html

2
https://www.fil.ion.ucl.ac.uk/spm/

3
http://surfer.nmr.mgh.harvard.edu/
