# Supplementary material for: Reconsideration of the DTI-ALPS index as a glymphatic surrogate: disease-specific findings in small vessel disease due to cerebral amyloid angiopathy or arteriolosclerosis
Source: Front Aging Neurosci. 2026 Sep 22;18:1933808. doi: 10.3389/fnagi.2026.1933808 (PMC13638668; doi:10.3389/fnagi.2026.1933808)
Supplement: Supplementary file 1 [file Data_Sheet_1.docx]

**Reconsideration of the DTI-ALPS index as a glymphatic surrogate: disease-specific findings in small vessel disease due to cerebral amyloid angiopathy or arteriolosclerosis**

**Supplementary data:**

Table S1. Detailed sequence parameters for 5.0T MRI.

Table S2. Disease-specific differences of the DTI-ALPS index and quantified PVS.

Table S3. Univariable regression analyses of clinical factors associated with the DTI-ALPS index

Table S4. Univariable regression analyses of imaging factors associated with the DTI-ALPS index

Figure S1. Flow chart of the study.

| Table S1. Detailed sequence parameters for 5.0T MRI. | | | |  |  |  |
| --- | --- | --- | --- | --- | --- | --- |
| Sequence | FOV | Matrix | Slice thickness | Gap | TR (ms) / TE (ms) | Scan Time |
| T1WI | 256 mm× 228 mm | 368× 368 | 1.0 mm | 0 | 9.1 / 2.5 | 4 min 00 s |
| T2WI | 256 mm× 228 mm | 368× 368 | 1.0 mm | 0 | 3000 / 399.64 | 4 min 03 s |
| FLAIR | 256 mm× 228 mm | 368× 368 | 1.0 mm | 0 | 6500 / 361.76 | 6 min 43 s |
| SWI | 220 mm× 185 mm | 624× 624 | 2.0 mm | 0 | 22.4 / 13.6 | 6 min 08 s |

Abbreviations: FLAIR: Fluid-Attenuated Inversion Recovery, FOV: field of view, MRI: magnetic resonance imaging, SWI: susceptibility-weighted imaging, T1WI: T1-weighted imaging, T2WI: T2-weighted imaging, TE: echo time, TR: repetition time.

| Table S2. Disease-specific differences of the DTI-ALPS index and quantified PVS | | | | |
| --- | --- | --- | --- | --- |
|  | Total （n=134） | CAA （n=56） | aCSVD （n=78） | *p* value |
| PVS quantitative measures | |  |  |  |
| JPVS count | 561.5 (246-1142) | 909 (400.5-1632.5) | 459 (186-882) | <0.001 |
| DWM-PVS count | 350 (164-648) | 525.5 (239.5-837.5) | 260.5 (92-545) | <0.001 |
| WM-PVS count | 972.5 (406-1853) | 1680 (799-2349) | 769.5 (311-1386) | <0.001 |
| JPVS volume | 646.9 (236.8-1728.0) | 1173.7 (440.6-2686.8) | 535.7 (179.8-1293.0) | 0.003 |
| DWM-PVS volume | 431.8 (152.8-912.5) | 613.6 (293.4-1105.0) | 322.5 (111.3-895.1) | 0.007 |
| WM-PVS volume | 1154.0 (432.3-2788.0) | 1739.5 (733.7-3756.1) | 937.5 (304.6-2146.4) | 0.003 |
| DTI-ALPS index | 1.17 ± 0.14 | 1.23 ± 0.13 | 1.12 ± 0.13 | <0.001 |

Abbreviations: aCSVD: arteriolosclerosis cerebral small vessel disease, DTI-ALPS: diffusion tension imaging analysis along perivascular space, CAA: cerebral amyloid angiopathy, PVS: perivascular space, DWM: deep white matter, JPVS: juxtacortical perivascular spaces, WM: white matter.

| Table S3. Univariable regression analyses of clinical factors associated with the DTI-ALPS index | | | | | |  |
| --- | --- | --- | --- | --- | --- | --- |
|  | Total (n=134) | | aCSVD (n=78) | | CAA (n=56) | |
|  | β (SE) | p value | β (SE) | p value | β (SE) | p value |
| Age | -0.00 (-0.003, 0.002) | 0.697 | -0.001 (-0.004, 0.002) | 0.453 | -0.002 (-0.006, 0.001) | 0.219 |
| Sex (female) | 0.08 (0.03, 0.13) | 0.001 | 0.10 (0.04, 0.16) | 0.001 | 0.06 (-0.02, 0.13) | 0.126 |
| Hypertension | -0.07 (-0.13, -0.02) | 0.012 | -0.01 (-0.10, 0.08) | 0.874 | -0.06 (-0.12, 0.01) | 0.144 |
| Diabetes | 0.03 (-0.03, 0.09) | 0.362 | 0.09 (0.02, 0.17) | 0.010 | -0.07 (-0.16, 0.03) | 0.158 |
| Hyperlipidemia | 0.03 (-0.02, 0.08) | 0.226 | 0.03 (-0.03, 0.09) | 0.252 | 0.02 (-0.05, 0.09) | 0.578 |
| Smoke | -0.07 (-0.11, -0.02) | 0.006 | -0.09 (-0.15, 0.04) | 0.001 | -0.03 (-0.10, 0.04)) | 0.351 |
| Drink | -0.08 (-0.13, -0.04) | <0.001 | -0.09 (-0.15, -0.03) | 0.001 | -0.06 (-0.13, 0.01) | 0.096 |
| Education | 0.004 (-0.04, 0.05) | 0.884 | 0.004 (-0.06, 0.06) | 0.884 | 0.01 (-0.06, 0.08) | 0.83 |

* General linear regression models were fitted with the DTI-ALPS index as the dependent variable.

Abbreviations: aCSVD: arteriolosclerosis cerebral small vessel disease, DTI-ALPS: diffusion tensor imaging analysis along perivascular space, CAA: cerebral amyloid angiopathy, CI: confidence interval.

| Table S4. Univariable regression analyses of imaging factors associated with the DTI-ALPS index | | | | | | |
| --- | --- | --- | --- | --- | --- | --- |
|  | Total (n=134) | | aCSVD (n=78) | | CAA (n=56) | |
|  | β (95% CI) | p value | β (95% CI) | p value | β (95% CI) | p value |
| ICH presence | 0.01 (-0.04, 0.06) | 0.722 | 0.004 (-0.07, 0.08) | 0.925 | 0.04 (-0.11, 0.03) | 0.217 |
| Lobar ICH | 0.05 (-0.01, 0.11) | 0.127 | - | - | 0.04 (-0.11, 0.03) | 0.217 |
| CMBs presence | -0.04 (-0.11, 0.03) | 0.269 | -0.06 (-0.15, 0.02) | 0.153 | -0.01 (-0.12, 0.09) | 0.785 |
| Strictly lobar CMBs | 0.08 (0.03, 0.14) | **0.002** | - | - | 0.02 (-0.05, 0.09) | 0.548 |
| cSS presence | 0.07 (0.01, 0.12) | **0.028** | - | - | -0.02 (-0.08, 0.05) | 0.639 |
| BG-PVS grades | 0.001 (-0.03, 0.03) | 0.968 | 0.02 (-0.02, 0.05) | 0.371 | 0.02 (-0.03, 0.06) | 0.445 |
| Lacune presence | -0.10 (-0.16, -0.05) | **<0.001** | -0.05 (-0.16, 0.06) | 0.335 | -0.06 (-0.13, 0.01) | 0.074 |
| Fazekas scores |  |  |  |  |  |  |
| PWMH | -0.08 (-0.11, -0.05) | **<0.001** | -0.09 (-0.14, -0.04) | **<0.001** | -0.04 (-0.09, 0.01) | 0.088 |
| DWMH | -0.05 (-0.08, -0.02) | **0.001** | -0.05 (-0.09, -0.01) | **0.017** | -0.03 (-0.07, 0.02) | 0.23 |
| Total | -0.04 (-0.06, -0.03) | **<0.001** | -0.04 (-0.06, -0.02) | **0.001** | -0.02 (-0.05, 0.003) | 0.084 |
| WMH volume^#^ | -0.07 (-0.09, -0.05) | **<0.001** | -0.05 (-0.09, -0.01) | **0.001** | -0.09 (-0.13, -0.05) | **0.001** |
| CSVD score | -0.04 (-0.07, -0.02) | **0.002** | -0.02 (-0.06, 0.01) | 0.189 | -0.03 (-0.07, 0.01) | 0.164 |
| BPF | 1.50 (0.83, 2.17) | **<0.001** | 1.19 (0.31, 2.07) | **0.010** | 1.64 (0.76, 2.52) | **0.001** |
| FA | 1.62 (1.13, 2.11) | **<0.001** | 1.25 (0.63, 1.88) | **<0.001** | 1.67 (0.88-2.46) | **<0.001** |
| MD, ×10^-3^ | -0.87 (-1.20, -0.54) | **<0.001** | -0.89 (-1.39, -0.40) | **0.001** | -0.71 (-1.11, -0.31) | **0.001** |
| PSMD, ×10^-3^ | -0.37 (-0.48, -0.25) | **<0.001** | -0.40 (-0.56, -0.24) | **<0.001** | -0.29 (-0.44, -0.14) | **<0.001** |

* General linear regression models were fitted with the DTI-ALPS index as the dependent variable.

^#^ Standardized transformed (Z-score).

Abbreviations: aCSVD: arteriolosclerosis cerebral small vessel disease, DTI-ALPS: diffusion tensor imaging analysis along perivascular space, BG: basal ganglia, BPF: brain parenchymal fraction, CAA: cerebral amyloid angiopathy, CI: confidence interval, CMB: cerebral microbleed, CSVD: cerebral small vessel disease, cSS: cortical superficial siderosis, DWMH: deep white matter hyperintensity, ICH: intracerebral hemorrhage, PVS: perivascular space, PWMH: periventricular white matter hyperintensity, PSMD: peak width of skeletonized mean diffusivity, WMH: white matter hyperintensity.

**Figure S1.** Flow chart of the study.

Abbreviations: CAA: cerebral amyloid angiopathy, CSVD: cerebral small vessel disease, DTI: diffusion tensor imaging, MRI: magnetic resonance imaging, T2WI: T2-weighted imaging.
